# Novel RNAseq-Informed Cell-type Markers and Their Regulation Alter Paradigms of *Dictyostelium* Developmental Control

**DOI:** 10.3389/fcell.2022.899316

**Published:** 2022-05-05

**Authors:** Gillian Forbes, Zhi-Hui Chen, Koryu Kin, Pauline Schaap

**Affiliations:** ^1^ School of Life Sciences, University of Dundee, Dundee, United Kingdom; ^2^ Institut de Biologia Evolutiva (CSIC-Universitat Pompeu Fabra), Barcelona, Spain

**Keywords:** cell-type specific RNAseq, spatio-temporal gene expression, morphogenetic signalling, cell fate mapping, cyclic diguanylate, differentiation inducing factor, cyclic AMP

## Abstract

Cell differentiation is traditionally monitored with a few marker genes, which may bias results. To understand the evolution and regulation of the spore, stalk, cup and basal disc cells in Dictyostelia, we previously performed RNAseq on purified cell-types of taxon-group representative dictyostelids. Using promoter-*lacZ* constructs in *D. discoideum*, we here investigate the spatio-temporal expression pattern of 29 cell-type specific genes. Genes selected for spore- or cup-specificity in RNAseq were validated as such by *lacZ* expression, but genes selected for stalk-specificity showed variable additional expression in basal disc, early cup or prestalk populations. We measured responses of 25 genes to 15 single or combined regimes of induction by stimuli known to regulate cell differentiation. The outcomes of these experiments were subjected to hierarchical clustering to identify whether common modes of regulation were correlated with specific expression patterns. The analysis identified a cluster combining the spore and cup genes, which shared upregulation by 8-bromo cyclic AMP and down-regulation by Differentiation Inducing Factor 1 (DIF-1). Most stalk-expressed genes combined into a single cluster and shared strong upregulation by cyclic di-guanylate (c-di-GMP), and synergistic upregulation by combined DIF-1 and c-di-GMP. There was no clustering of genes expressed in other soma besides the stalk, but two genes that were only expressed in the stalk did not respond to any stimuli. In contrast to current models, the study indicates the existence of a stem-cell like soma population in slugs, whose members only acquire ultimate cell fate after progressing to their terminal location during fruiting body morphogenesis.

## Introduction

Multicellularity allowed organisms to set aside a proportion of non-propagating cells that support, protect and feed the propagating cells, i.e. the spores or germ cells. The evolution of complexity in multicellular organisms involved the specialization of these non-propagating somatic (soma = body) cells into a progressively larger number of phenotypically distinctive cell types. While the evolution of novel genes has undoubtedly contributed to ongoing cell-type specialization, the differential up- or down regulation of existing genes played a major role in generating cells specialized in tasks like secretion, support, motility, metabolism and transport that were already displayed by the unicellular ancestor. A main feature of embryonic development is therefore the regulation of cell-type specific gene expression at the appropriate time and location within the organism. Communication between cells using molecules that are either secreted or exposed on the cell surface is the most common mode of regulating gene expression.

Mechanisms controlling cell differentiation were traditionally studied using the specialized phenotype as a marker, or an aspect thereof, such as an enzyme activity. However, advances in molecular biology made it possible to study this process much earlier at the level of gene transcription, using one or a few cell-type specific genes as markers. Recent advances in genomics and single cell transcriptomics have highlighted that even cells of a specific type show heterogeneity in gene expression (Hauser et al., 2020; Tan and Wilkinson, 2020), indicating that results obtained with one or a few marker genes may represent an oversimplification. While using transcriptomics for every experiment investigating cell differentiation would be a costly and laborious affair, the methodology does provide us with the opportunity to select a broader representative range of marker genes.

We investigate the mechanisms and evolution of cell-type specialization in Dictyostelia. These soil amoebas feed on bacteria as single cells but come together when starving to form a multicellular fruiting body (sorocarp) with resilient spores. They can be subdivided into four major groups, of which groups 1, 2 and 3 only differentiate into one somatic cell type, the stalk cells that support the spore mass. However, group 4, which contains the model *D. discoideum,* underwent innovations such as transformation of the aggregate into an intermediate migrating slug and differentiation of two more somatic cell types, the disc and cup cells, which anchor the stalk to the substratum and the spore mass to the stalk, respectively. Prespore differentiation initiates in the posterior three quarters of the slug; the anterior quarter will form the stalk, while the basal disc and cup cells are derived from so-called anterior-like cells (ALCs) that are scattered throughout the prespore region but are more similar to prestalk cells (Sternfeld and David, 1982). Variability in the pre-aggregative cell population in e.g. cell cycle phase predisposes cells to either prespore or somatic cell fate (Weijer et al., 1984; Gomer and Firtel, 1987; Ohmori and Maeda, 1987; Wang et al., 1988a), supposedly by altering their sensitivity to differentiation inducing signals (Thompson and Kay, 2000). Several secreted or exposed signals have been identified that affect cell differentiation, but a comprehensive understanding of the developmental programme has yet to be achieved (Loomis, 2014; Consalvo et al., 2019; Kawabe et al., 2019).

The signalling processes leading to spore formation are best understood. Cells start to express spore coat genes just after aggregation and to prefabricate spore wall materials in Golgi-derived vesicles. cAMP plays a dominant role in the induction of spore coat gene expression. Due to the activity of mainly adenylate cyclase G (AcgA) (Alvarez-Curto et al., 2007), both intracellular and extracellular cAMP increase in the slug prespore region. This results in the combined activation of cell surface cAMP receptors (cARs) and intracellular cAMP dependent protein kinase (PKA), which both combine to induce prespore differentiation (Schaap and Van Driel, 1985; Hopper et al., 1993). During fruiting body formation, secreted signals such as SDF-2 (spore differentiation factor 2) and discadenine act to further increase cAMP and activate PKA, which triggers spore maturation and expression of genes that control spore dormancy and germination (Richardson and Loomis, 1992; Mann et al., 1994; Wang et al., 1999; Anjard and Loomis, 2008; Kin et al., 2018).

The regulation of the different somatic cell types is mainly deduced from the expression patterns of two extracellular matrix genes, *ecmA* and *ecmB,* that were originally identified as transcripts upregulated by DIF-1 (Jermyn et al., 1987)*,* a secreted factor that induces differentiation of vacuolated stalk-like cells *in vitro* (Morris et al., 1987). Both *ecmA* and *ecmB* have complex expression patterns that are regulated by different regions of their promoters. The full *ecmA* promoter directs expression in the slug prestalk cells and ALCs, and in the stalk, lower cup and basal disc of the fruiting body (Jermyn and Williams, 1991). The cap-site proximal pstA region of the *ecmA* promoter directs expression in the front half of the prestalk region and the stalk, while the more distal pstO sequence directs expression in ALCs and the upper cup (Early et al., 1993). In the slug, the full *ecmB* promoter directs expression in the core of the tip and in a subpopulation of ALCs, the pstB cells, while in the fruiting body the *ecmB* promoter is active in the stalk, basal disc and upper and lower cups (Jermyn and Williams, 1991). The cap proximal *ecmB_ST* region lacks upper cup expression (Ceccarelli et al., 1991). *In situ* hybridization patterns of genes enriched in slug prestalk cells both overlapped with the expression pattern of *ecmA* and *ecmB,* or showed novel configurations, indicative of extensive heterogeneity of the prestalk population (Maeda et al., 2003; Yamada et al., 2005). In addition, other prestalk-enriched genes were identified, such as several actin genes (Tsang et al., 1982), 2H-6 and 16-G1 (*cprB*) (Mehdy et al., 1983), PL1, D11 (*ampA*) and D14 (Barklis and Lodish, 1983), rasC (Reymond et al., 1984) and car2 (Saxe III et al., 1996). These genes mostly accumulate during aggregation and are inducible by cAMP and not by DIF-1. It was reported that these cAMP-inducible prestalk genes were less prestalk-enriched than the DIF-1 induced prestalk genes (Jermyn et al., 1987) and they were therefore not as widely used.

While DIF-1 was put forward as the main inducer of prestalk and stalk differentiation (Morris et al., 1987; Williams et al., 1987), mutants defective in DIF-1 biosynthetic enzymes, such as the polyketide synthase StlB, were more recently found to still form the stalk, but not the basal disc and part of the lower cup (Saito et al., 2008). The basal disc also consists of stalk-like vacuolated cells, suggesting that the stalk-like cells that are induced by DIF-1 *in vitro* are basal disc cells. Stalk formation does not occur in mutants that lack the diguanylate cyclase, *dgcA*, which is expressed throughout the prestalk region. DgcA synthesises c-di-GMP which induces stalk cell differentiation in *D. discoideum* V12M2 *in vitro* (Chen and Schaap, 2012). C-di-GMP acts by hyperactivating the adenylate cyclase AcaA, which is preferentially expressed at the slug tip, resulting in PKA activation and stalk formation (Chen et al., 2017). It is far from clear how the different populations of somatic cells in *Dictyostelium* are regulated and most studies have been focussed on *ecmA*, *ecmB* and their promoter subregions. Apart from yielding spore and stalk precursors, the slug cells and particularly those of the prestalk region have many other functions, such as the synthesis of the elastic slug sheath, providing motive force, chemotactic signalling, phototaxis, thermotaxis and directional cell movement. These functions likely require the transient expression of many genes that are not necessarily important for ultimate cell fate.

To increase the repertoire of cell type markers that are relevant for terminal cell differentiation, we recently performed transcriptome analysis of purified stalk, spore, cup and vegetative cells (Kin et al., 2018). For 12 genes with highly enriched expression in stalk, spore or cup cells, cell type specific expression was validated by fusion of their promoters to the β-galactosidase (*lacZ*) reporter gene and visualization of β-galactosidase activity in developing structures. Of this set two out of two spore-enriched genes and two out of three cup-enriched genes were only expressed in spore or cups, respectively. However only two out of seven stalk-enriched genes were stalk-specific, with the rest also being expressed in other somatic cell types. In this work, we further expanded the search for genes that are specific to the different somatic cell types. Using a subset of 25 cell-type marker genes we additionally investigated whether genes that share similar expression patterns also show the same modes of regulation by currently known developmental signals. Our results indicate the presence of a largely unspecified soma population throughout most of development and missing signals for specification of stalk and cup cell fate.

## Methods

### Cell Culture and Development

*D. discoideum* Ax2 was grown in HL5 axenic medium (Formedium), which was supplemented with 20–200 μg/ml G418 for transformed cell lines. For a histochemical β-galactosidase assay, post-vegetative cells were plated at 10^6^ cells/cm^2^ on nitrocellulose filters, supported by non-nutrient (NN) agar (1.5% agar in 8.8 mM KH_2_PO_4_ and 2.7 mM Na_2_HPO_4_) and incubated at 22°C until the desired developmental stage had been reached.

### DNA Constructs

The full 5′intergenic regions were amplified for each gene from Ax2 genomic DNA using the oligonucleotide primers listed in Supplementary Table S1 and OneTaq^®^ Hot Start (NEB), MyTaq (Bioline) or KOD (Merck) DNA polymerase. Routinely, XbaI and BglII sites were incorporated into the primer design and after digestion the PCR products were cloned into XbaI/BglII digested vector pDdgal-17 (Harwood and Drury, 1990), fusing the start codon and usually the first few amino-acids of the inserted gene (Supplementary Table S1) in frame with the *LacZ* coding sequence. When the promoter sequence contained XbaI and/or BglII, KpnI, NheI or BamHI were alternatively used. All constructs were validated by DNA sequencing and transformed into *D. discoideum* Ax2 by electroporation. Transformants were selected at 20 μg/ml G418, which was raised to 200 μg/ml G418, when cells expressed the reporter genes only weakly.

### Histochemical β-galactosidase Assay

Filters with developing structures were transferred to Whatman 3 MM chromatography paper soaked in 0.5% glutaraldehyde in Z buffer (60 mM Na_2_HPO_4_, 40 mM NaH_2_PO_4_, 10 mM KCl and 1 mM MgSO_4,_ pH 7.0) and incubated in a sealed chamber for 6 min. Structures were next fully submersed in 0.5% glutaraldehyde for 3 min. After washing with Z-buffer, structures were stained with X-gal as described previously (Dingermann et al., 1989). Staining times varied between genes, but different developmental stages of cells transformed with the same construct were stained for the same period.

### Gene Induction Experiments

Cells were developed at 2.5 × 10^6^ cells/cm^2^ on NN agar to a stage just before the gene started to be expressed. This varied from loose aggregates (*ecmA*) to tipped mounds or first fingers (most genes). Structures were disrupted with a rubber scraper, harvested in stalk salts (10 mM KCl, 2 mM NaCl and 1 mM CaCl_2_ in 10 mM MES, pH 6.2) and dissociated into single cells by passing through a 23-gauge needle. Cells were diluted to 5 × 10^5^ cells/ml or to 5 × 10^6^ cells/ml for poorly expressed genes and incubated in a total volume of 100 µL with various stimuli, such as 1–10 µM c-di-GMP (BioLog), 100 nM DIF-1 (Enzo), 0.3 mM cAMP (Sigma), 10 mM 8Br-cAMP (BioLog), 7.5 mM DMO (5,5-dimethyl-2,4-oxazolidinedione) and 30 mM BHQ (2,5,-di-(tert-butyl)-1,4-hydroquinone). Cells were incubated while shaken at 22°C and 1,000 rpm on a micoplate shaker for 8 h and then frozen at -80°C, or frozen directly at t = 0 h. All experiments were performed at least three times with three replicates each.

### Spectrophotometric β-galactosidase Assay

Cells were lysed by three cycles of freeze-thawing, with thawing under vigorous shaking. Lysates were supplemented with 10 µL of 40 mM CPRG (chlorophenolred-b-D-galactopyranoside, Roche) dissolved in 2.5 x PBSA (150 mM Na_2_HPO_4_, 100 mM NaH_2_PO_4_, 25 mM KCl and 2.5 mM MgSO4) and 30 µL of 2.5 x ZM-buffer (1% mercaptoethanol in 2.5 x PBSA) and incubated at 22°C. OD_574_ was measured at regular intervals using a microplate spectrophotometer. From a period where OD_574_ increased linearly, ΔOD/min was calculated by dividing the difference in OD values between two time points by the elapsed time. Data were standardized as percentage of the ΔOD/min value obtained after incubation with 3 µM c-di-GMP, which induced a reasonable level of expression for most tested genes or as fold-change relative to control (no addition). The ΔOD/min and standardized values for all experiments are archived in Supdata2_Induction.xlsx.

### Statistical Analysis

Basic descriptive statistics (Means, SD and SE) of the gene induction data were computed in the data spreadsheet Supdata2_Induction.xlsx. To assess significant differences between the experimental data, data standardized as percentage of 3 µM c-di-GMP for each gene were analysed by ANOVA (analysis of variance) in Sigmaplot (http://sigmaplot.co.uk/). Since several sets contained data that were not normally distributed, Kruskal–Wallis one-way ANOVA on ranks was used to identify significant differences between treatments. *p*-values for pair-wise comparison of treatment data were determined by both the stringent Tukey and less stringent Student-Newman-Keuls test. The outcomes for both tests are listed with the experimental data for each gene in Supdata2_Induction.xlsx. *p*-values for biologically relevant comparisons are summarized in Supplementary Figure S4 and gene induction fold-changes between treatments, annotated with *p*-values are shown in Supplementary Figure S5.

Hierarchical clustering of averaged induction by different treatments for each gene was performed in the datamining software Orange (Demsar et al., 2013). Distances between the responses of each gene to the different treatments were determined by Pearson correlation. Hierarchical relations between the clusters were determined by average linkage. The data matrices and results of the analyses are archived in SupData3_Clusteranalysis.xlsx.

## Results

### Selection of Novel Cell-Type Marker Genes

Genomes and developmental transcriptomes are currently available for five *Dictyostelium* species that span the four major taxon groups (Eichinger et al., 2005; Parikh et al., 2010; Heidel et al., 2011; Gloeckner et al., 2016). Transcriptome data are also available for the purified prestalk and prespore cells of the group 4 species *Dictyostelium discoideum* (*Ddis*) and *D. purpureum* (*Dpur*) (Parikh et al., 2010) and for purified spore, stalk, cup and vegetative cells of *Ddis* (Kin et al., 2018) as well as the spore, stalk and vegetative cells of the group 3 species *D. lacteum* (*Dlac*) (Forbes et al., 2019) and spores and stalk cells of the group 2 species *Polysphondylium pallidum* (*Ppal*) (Heidel et al., 2011). These studies allowed us to identify novel cell type markers in *Ddis* and to investigate to what extent the genes and their expression patterns are conserved throughout Dictyostelia.

We first sought to expand the range of markers identified previously (Kin et al., 2018) focussing on genes that were specific to either of the *Ddis* mature cell types and upregulated in development, the latter to avoid genes that become cell-type specific by downregulation in other cells. We also favoured genes that were conserved more broadly across the Dictyostelid phylogeny. Note that in the earlier study we could not obtain basal disc specific transcripts since the disc is attached to the stalk by shared cellulose walls (Kin et al., 2018). To effectively visualize gene expression and measure its regulation, we also favoured genes with high RNAseq read counts, although it was often not possible to optimize all preferred features simultaneously.

**FIGURE 1:**
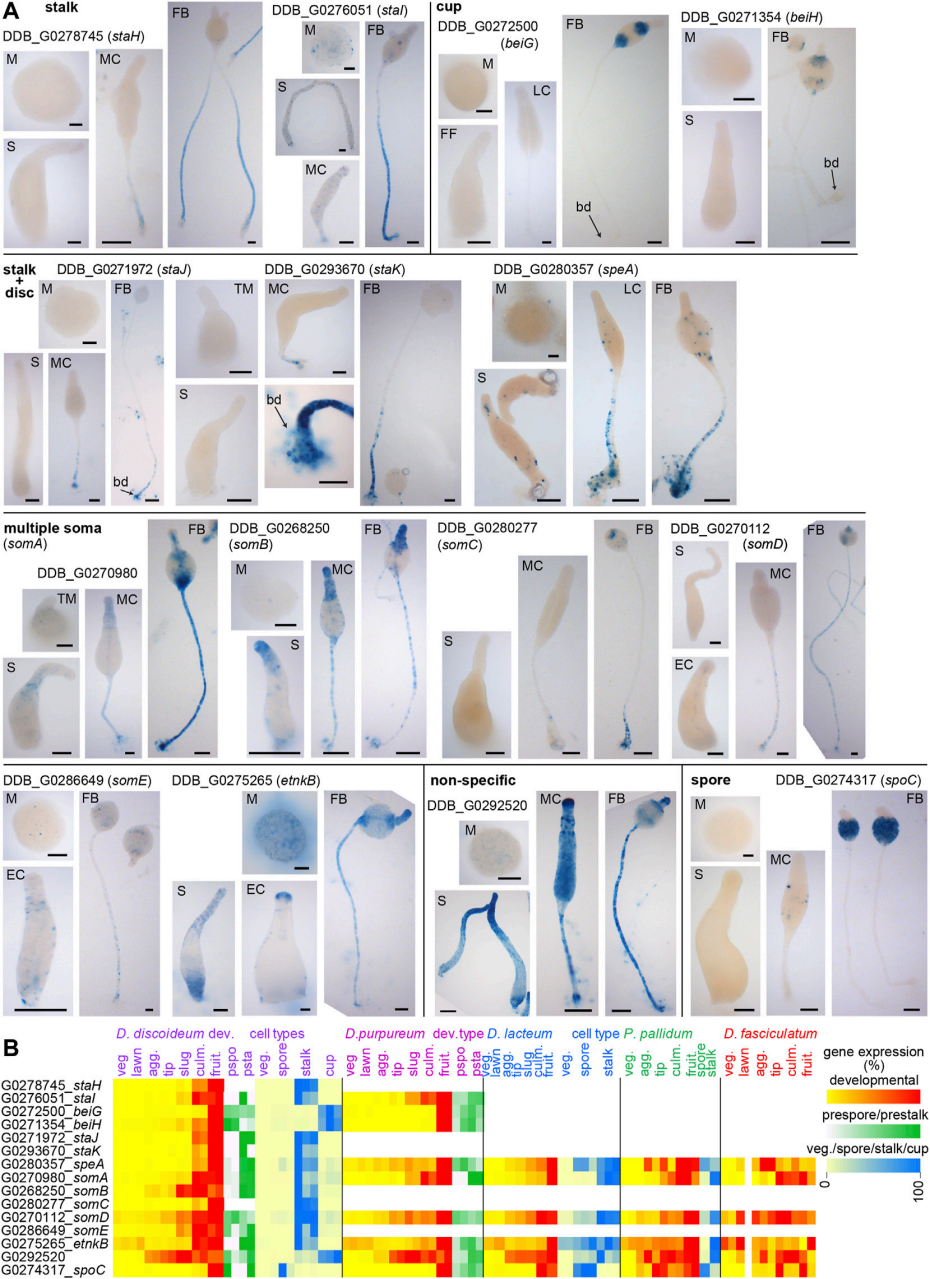
Expression patterns of putative cell type specific genes. **(A)** *. Spatial patterns. Ddis* AX2 cells were transformed with fusion constructs of *LacZ* and the 5′ intergenic regions of putative cell-type specific genes identified from cell-type specific RNAseq (Kin et al., 2018). Cells were starved at 10^6^ cells/cm^2^ on nitrocellulose filters supported by non-nutrient agar to induce multicellular development. Structures were fixed and incubated with X-gal to visualize β-galactosidase activity at the mound (M), first finger (FF), slug (S), culminant (EC: early culminant, MC: mid-culminant, LC: late culminant) and mature fruiting body (FB) stages. Per transformant, the incubation period was the same for all stages. Bars: 50 µm. **(B)**. *Transcriptomes*. RNAseq data for the investigated genes were obtained from published experiments of developmental time courses and purified cell types across taxon group representative species (Parikh et al., 2010; Heidel et al., 2011; Gloeckner et al., 2016) (Kin et al., 2018). Normalized read counts are standardized to maximum or summed (prestalk/prespore) read counts per series and shown as heatmaps of two or three replicate experiments (veg: feeding cells, lawn: starving cells, agg: aggregate, tip: tipped mound, culm. culminant, fruit. fruiting body, pspo. prespore, psta. prestalk).

In total we selected 15 novel *Ddis* cell type markers from the parent study (Kin et al., 2018), of which 6 were conserved throughout the dictyostelid phylogeny and six were restricted to group 4 or branch II (Supplementary Figure S1). The expression patterns of the genes were validated by transforming *Ddis* with *lacZ* fused to the 5′intergenic region of the genes, followed by visualization of β-galactosidase activity in developing structures (Figure 1A). Developmental and cell-type specific expression data of the genes and their orthologs across Dictyostelia are shown in Figure 1B.

Two of the three selected putative cup genes and the putative spore gene were specific to the cup and spore regions, respectively. The remaining cup gene DDB_G0292520 was also expressed in the stalk and prespore region and is therefore classified as non-specific. Of the 11 selected putative stalk genes, two were specific to the stalk, three were additionally expressed in the basal disc, while the remaining genes were also expressed in the cup region. For two genes in this set DDB_G0270112 (*somD*) and DDB_G0286649 (*somE*) β-galactosidase activity was very weak, even after selection at 200 μg/ml G418.

Most genes were poorly or not expressed in mounds and slugs as was also evident from their developmental expression profile (Figure 1B). They appear to differ in this respect from the DIF-induced stalk markers *ecmA* and *ecmB,* which show considerable expression in the prestalk and anterior-like cells in slugs, see e.g. (Jermyn et al., 1996). In this study, all developmental stages from the same species were stained with X-gal equally long, i.e. the time needed for one of the stages to show intense staining. When using this approach, Ax2 cells transformed with ecmA-gal (Jermyn and Williams, 1991) or ecmA-ile-gal, with reduced β-galactosidase stability (Detterbeck et al., 1994), showed less intense but visible staining in slugs compared to culminants, while ecmB-gal slugs showed only very light staining at the tip (Supplementary Figure S2). Concurrently, *ecmA* shows significant and *ecmB* little expression in slugs in RNAseq experiments (Supplementary Figure S3B). The newly identified markers DDB_0270980 (*somA*), DDB_G0268250 (*somB*), DDB_G0286649 (*somE*) and *etnkB* resemble *ecmA* in also showing expression in slugs (Figure 1A), as is also evident from RNAseq data (Figure 1B).

For the newly identified marker genes, we replaced their 12-character locus tags by more mnemonic gene names. Our preference is to name genes after protein function, but as this is mostly unknown, genes expressed in stalk or stalk + disc were named *staH-staK*, those specific to cups and spores were named *beiG/beiH* and *spoC,* respectively, while genes expressed in stalk, disc and cups, i.e. in all somatic cells, were named *somA-somE* (Table 1).

**TABLE 1:** Gene expression patterns, conservation and function.

Locus tag	*Name*	Pattern	Conservation	Function	Domains
DDB_G0278745*	*staH*	st	grp 4	Unknown	DUF829
DDB_G0276051*	*staI*	st	grp 4	Unknown	H-lectin
DDB_G0269904^#^	*staG*	st	*Ddis*	Unknown	none
DDB_G0277757^%^	*staD*	st + p	all grps	Unknown	CBM49
DDB_G0271972*	*staJ*	st + d	*Ddis*	Unknown	signal peptide
DDB_G0293670*	*staK*	st + d	grp 4	unknown	Methyltransf_23
DDB_G0280357*	*speA*	st + d	all grps	unknown	spy1
ecmB_ST^$^	*ST*	st + d			
DDB_G0288331^#^	*expl7*	st + d	all grps	expansin	DPBB_1
ecmA_pstA^£^	*pstA*	st + d			
DDB_G0279361^#^	*staF*	ms	grp 4	unknown	signal peptide
DDB_G0287091^#^	*staE*	ms	grp 4	unknown	none
DDB_G0269132^&^	*ecmB*	ms	grp 4	matrix protein	Dicty_CTDC
DDB_G0280277*	*somC*	ms	*Ddis*	unknown	CBM49
DDB_G0270980*	*somA*	ms + p	all grps	unknown	claudin-2
DDB_G0280217^#^	*somF*	ms + p	*Ddis*	unknown	none
DDB_G0271196^%^	*staC*	ms + p	*Ddis*	unknown	Dicty_CAD
DDB_G0277853^&^	*ecmA*	ms + p	grp 4	matrix protein	Dicty_CTDC
ecmA_pstO^£^	*pstO*	ms + p			
DDB_G0272500*	*beiG*	cup	grp 4, *Pvio*	unknown	PAP2
DDB_G0271354*	*beiH*	cup	grp 4	unknown	signal peptide
DDB_G0276687^#^	*beiE*	cup	*Ddis*	hssA-related	none
DDB_G0271780^#^	*beiF*	cup	*Ddis*	unknown	ADH_N
DDB_G0274317*	*spoC*	spo	all grps	unknown	none
DDB_G0288489^#^	*spoA*	spo	all grps	unknown	none
Pattern only					
DDB_G0268250*	*somB*	ms + p	grp 4	unknown	signal peptide
DDB_G0270112*	*somD*	ms	all grps	unknown	none
DDB_G0286649*	*somE*	ms + p	*Ddis*	unknown	CBM49
DDB_G0275265*	*etnkB*	ms + p	all grps	ethanolamine kinase B	Choline_kinase
DDB_G0292520*		ns	all grps	unknown	none
DDB_G0267476^#^	*sigK*	ms + p	grp 4,3	unknown	Laminin_EGF
DDB_G0295797^#^		ms + p	all grps	unknown	none
DDB_G0285289^#^	*spoB*	spo	all grps	unknown	none
DDB_G0275745^#^	*tgrR1*	ms + p	grp 4	unknown	signal peptide

Initial studies of the genes are indicated by locus tag superscripts: * studied here; # (Kin et al., 2018); %:(Chen et al., 2017); $: (Ceccarelli et al., 1991); £:(Early et al., 1993); &:(Jermyn and Williams, 1991). For *StaE* basal disc expression was initially not reported (Kin et al., 2018), but was detected in replicate experiments (Supplementary Figure S3).

Locus tags below the "Pattern only" heading were not analysed for gene induction. Abbreviations: s: stalk only; s + d: stalk + basal disc; ms: all mature soma; ms + p: mature soma + prestalk; spo: spores; ns: non-specific; grp: taxon group; *Pvio: Polyspondylium violaceum.*

Of the cup, stalk or stalk + disc specific genes, BLASTp query and phylogenetics showed that only one, the stalk + disc gene *speA* (speedyA, DDB_G0280357), was conserved throughout Dictyostelia (Supplementary Figure S1), although in taxon groups 2 and 3, *speA* was also expressed in spores (Figure 1B). The others, except *staJ* (DDB_G0271972), were however more broadly conserved in group 4 (Supplementary Figure S1). SpoC (DDB_G0274317) was conserved and spore-specific throughout Dictyostelia. Of the stalk + cup + disc set, *somA* (DDB_G0270980) was present and stalk-enriched throughout Dictyostelia as well as *somD* (DDB_G0270112) and *etnkB* (DDB_G0275265), although the latter was not stalk-enriched in group 2 (Figures 1B; Supplementary Figure S1).

In the parent study, we analysed spatial expression patterns of 12 putative cell-type specific genes (Kin et al., 2018). While not selected for evolutionary conservation, seven of those genes were more broadly conserved nevertheless, inclusive of their cell-type specificity (Supplementary Figure S3). Altogether, the novel cell type markers provide the opportunity to investigate cell-type specialization in a much broader evolutionary context than before.

### Regulation of Cell Type Marker Genes by Secreted Signals

**FIGURE 2:**
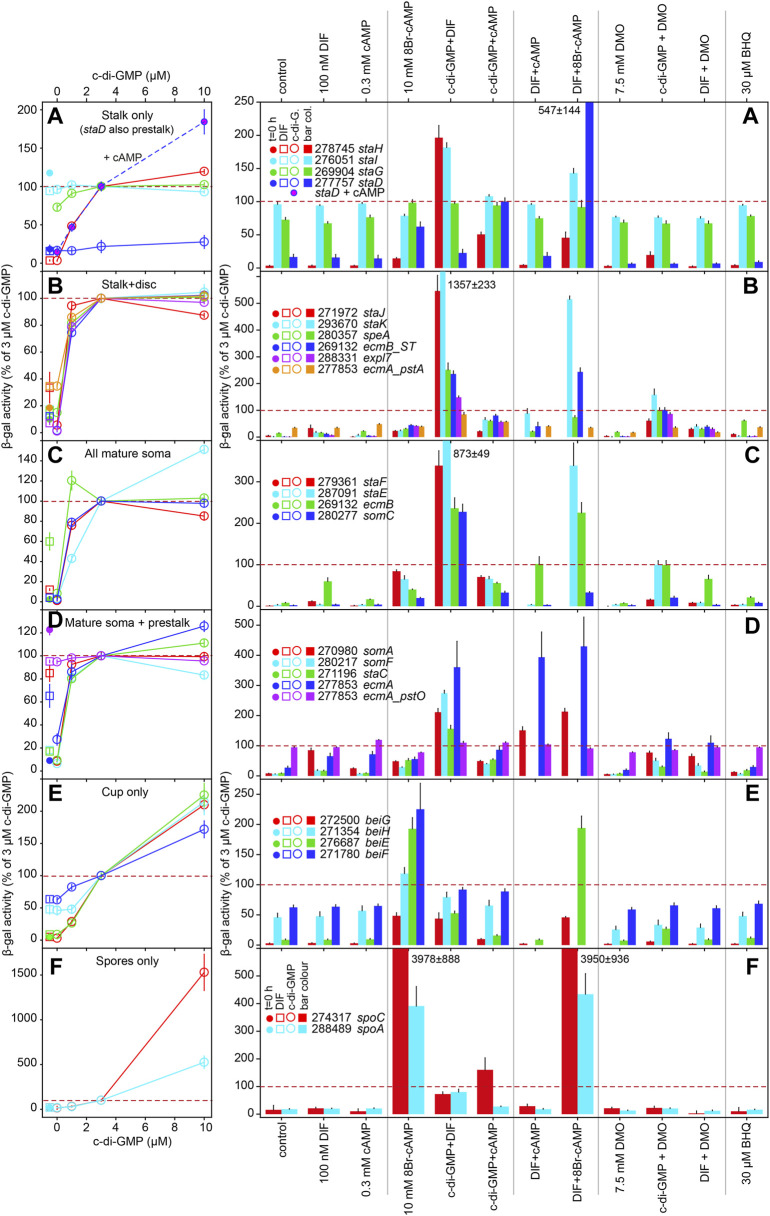
Regulation of cell-type marker genes by signal molecules. AX2 cells transformed with promoter-lacZ fusions were developed to aggregates or tipped mounds/first fingers. Structures were dissociated and incubated in stalk salts for 8 h with the indicated compounds and spectrophotometrically assayed for β-galactosidase activity. Experiments were performed at least three times in triplicate. Data are expressed as percentage of activity obtained after incubation with 3 µM c-di-GMP and are shown as means and SE. Since induction by 3 µM c-di-GMP is therefore 100% for all genes by definition, we represent this value by a dashed red line. Missing bars represent genes not treated with some compounds. Dual stimulation regimens used the same concentrations of compounds as single stimulation and 3 µM for c-di-GMP. All experimental data with tests for significant differences between treatments are listed in SupData2_Induction.xlsx. The induction data for the 26 cell type markers are subdivided over 6 panels **(A-F)**, that each combine markers with similar spatial expression. For *staD* in panel A, dose-response effects for c-di-GMP were measured both in the absence (open circles) and presence of 0.3 mM cAMP (pink-filled circles), while *staD* data in the bar graph were expressed as percentage of 3 µM c-di-GMP + 0.3 mM cAMP. The left panels contain β-galactosidase values before stimulation (t = 0 h, closed circles) and after stimulation with 100 nm DIF (open squares) to the left of the c-di-GMP dose response curves. Genes are listed by locus tag minus the DDB_G0 prefix and by gene names.

Out of the 29 novel RNAseq informed cell-type markers that were validated here and previously (Kin et al., 2018) (Table 1), we selected 18 genes that showed expression patterns representative for the whole set to study regulation by added stimuli. This subset was supplemented with *ecmA* and *ecmB* expressed from their full promoters (Jermyn and Williams, 1991) and from the pstO and pstA regions for *ecmA* (Early et al., 1993) and the ST-region for *ecmB* (Ceccarelli et al., 1991), and with the c-di-GMP responsive stalk gene *staC* and unresponsive gene *staD*, that were both detected in an RNAseq study of a *dgcA* knock-out (Chen et al., 2017). Pools of Ax2 cells, freshly transformed with gene fusions of *lacZ* to the promoters of all markers were developed on non-nutrient agar to a stage just before the gene in question started to be expressed. Structures were dissociated into single cells and incubated for 8 h with additives in microtiterplate wells. Incubation was at low cell density under vigorous agitation to reduce effects of endogenous signalling or cell-cell interactions. After cell lysis, β-galactosidase activity was measured spectrophotometrically in the same wells. The minimal preparative handling in this protocol (compared to e.g. RT-qPCR) allows for accurate quantitative assay of relative levels of gene expression in large numbers of samples (Schaap et al., 1993). The data were standardized as percentage of expression induced by 3 µM c-di-GMP (red dotted lines in Figure 2), which induced a fair level of β-galactosidase expression for almost all marker genes. Data were also standardized as fold-change relative to control (no addition), but since this was often very low as well as variable, it gave rise to disproportionate inflation of apparent induction in some experiments. The original and standardized data for each gene are listed in SupData2_induction.xlsx together with statistical evaluation (ANOVA on ranks) of significant differences between treatments. A summary of *p*-values for biologically relevant comparisons between treatments is shown in Supplementary Figure S4 and fold-change differences between treatments, annotated with *p*-values in Supplementary Figure S5.

The tested variables include the stalk inducers DIF-1 (Morris et al., 1987) and c-di-GMP, with the latter tested at 1, 3 and 10 µM to evaluate the previously observed dose dependency on stalk and cup gene expression (Chen et al., 2017), cAMP, with variable effects on different stalk genes (Berks and Kay, 1990; So and Weeks, 1994), the membrane-permeant PKA activator 8Br-cAMP, which promotes spore, stalk and cup cell maturation (Richardson et al., 1991; Takeuchi et al., 1994; Chen et al., 2017), the weak acid DMO and the Ca^2+^-ATPase inhibitor BHQ, which decrease intracellular pH and raise Ca^2+^ respectively, two processes reported to mediate or at least promote DIF-1 effects on stalk cell differentiation (Gross et al., 1983; Wang et al., 1990; Kubohara and Okamoto, 1994; Schaap et al., 1996). For comprehensive presentation the marker genes were subdivided into six classes, presented in panels A-F of Figure 2, with genes expressed in stalk, cup or spores only in panels A, E and F, genes expressed in the stalk and basal disc in panel B, genes expressed in all mature soma (stalk, disc and cups) in panel C, and genes expressed in mature soma and prestalk populations in panel D.

The left panels of Figure 2 show induction of the six classes of marker genes by increasing concentrations of c-di-GMP, compared to induction by 100 nM DIF-1. Of the 18 tested markers with stalk expression, 15 showed upregulation by c-di-GMP. The *ecmA-pstO* promoter and the stalk-only genes *staI, staG* and *staD* showed no or less than 2-fold upregulation (*staG*), respectively. *StaI* showed a 2-fold induction by combined c-di-GMP and DIF stimulation only, while *StaD* was uniquely upregulated by combined c-di-GMP and cAMP stimulation. The remaining stalk-only gene *staH* was 52-fold upregulated by 3 µM c-di-GMP. For the other 15 stalk markers, induction saturated at 1–3 µM c-di-GMP with half-maximal induction occurring between 0.1 and 0.5 µM c-di-GMP. The cup and spore only markers were also upregulated by c-di-GMP, but induction did not saturate at even 10 µM c-di-GMP (Figures 2E, F), with more extensive dose response curves estimating half-maximal activation of cup genes at 10 µM (Chen et al., 2017). The spore genes required similar to higher c-di-GMP concentrations than the cup genes for induction.

DIF-1 induced no expression of stalk-only genes (Figure 2A) and only some induction of stalk + disc genes, maximally reaching 40% of c-di-GMP induction for *staJ* (Figure 2B). For the mature soma genes (Figure 2C), only DIF induction of *ecmB* reached 60% of c-di-GMP induced levels, while for the mature soma + prestalk genes, DIF induction almost equalled c-di-GMP induction for *somA.* Strikingly, even for stalk genes that are not or poorly upregulated by DIF-1, there is a strong synergistic response to combined DIF-1 and c-di-GMP stimulation. For some genes like *somA* (Figure 2D, right panel), the effect is merely additive, but for *staJ, staK, staF, staE* and *ecmA* the effect of both stimuli far exceeds that of the two alone. DIF-1 also synergizes with 8Br-cAMP, but mostly to a lesser extent than with c-di-GMP. This could be due to suboptimal permeation and PKA activation by 8Br-cAMP, or by an inhibitory effect of 8Br-cAMP acting on cARs. For cup and spore genes, c-di-GMP induction is repressed by DIF-1, but 8Br-cAMP is an effective inducer, in line with the hypothesis that c-di-GMP also in these cells modestly stimulates PKA.

Compared to other stimuli cAMP has little effect on stalk, cup and spore gene expression by itself, but inhibits c-di-GMP induced expression of most stalk genes up by about 50%, except for *staD*, which is only induced by c-di-GMP in the presence of cAMP (Figure 2A). cAMP also inhibits c-di-GMP induction of two cup genes *beiG* end *beiE* and one of the spore genes (*spoA*). The effects of cAMP on DIF-1 induced expression are small and rather variable, except for *ecmA* where cAMP strongly upregulates DIF-1 induction (or *vice versa*). For the soma genes shown in Figures 2C, D, it is unclear how or in what proportion the expression of the gene in stalk, disc or cup cells contributes to the measured β-galactosidase activity. This may contribute to the variation in the responsiveness of the assayed genes to the added stimuli.

The effects of DMO, DIF-1 and BHQ are rather small compared to the large effects of c-di-GMP and 8Br-cAMP in Figure 2 and were therefore also presented separately as fold-change relative to control in Supplementary Figure S6. No marked effects of either DMO or BHQ were observed on spore and cup gene expression or from combined DMO and DIF treatment (Figures 2E, F, Supplementary Figure S6). However, DMO suppressed c-di-GMP induction of cup and spore genes from 40 to 90%. DMO alone has little effect on stalk genes, but stimulates DIF-1 induction o*f staK, ST, expl7* and *ecmA* about 4-fold. Effects of DMO on c-di-GMP induction of stalk genes are, if anything, somewhat inhibitory. The stalk genes *staJ, staK, speA, expl7, staF, ecmB, somC* and *staC* are over 2-fold up-regulated by BHQ. However, most of the relatively small effects of DMO or BHQ do not reach statistical significance with the stringent Tukey test (Supplementary Figure S5).

### Cluster Analysis of Gene Regulation by External Stimuli

**FIGURE 3:**
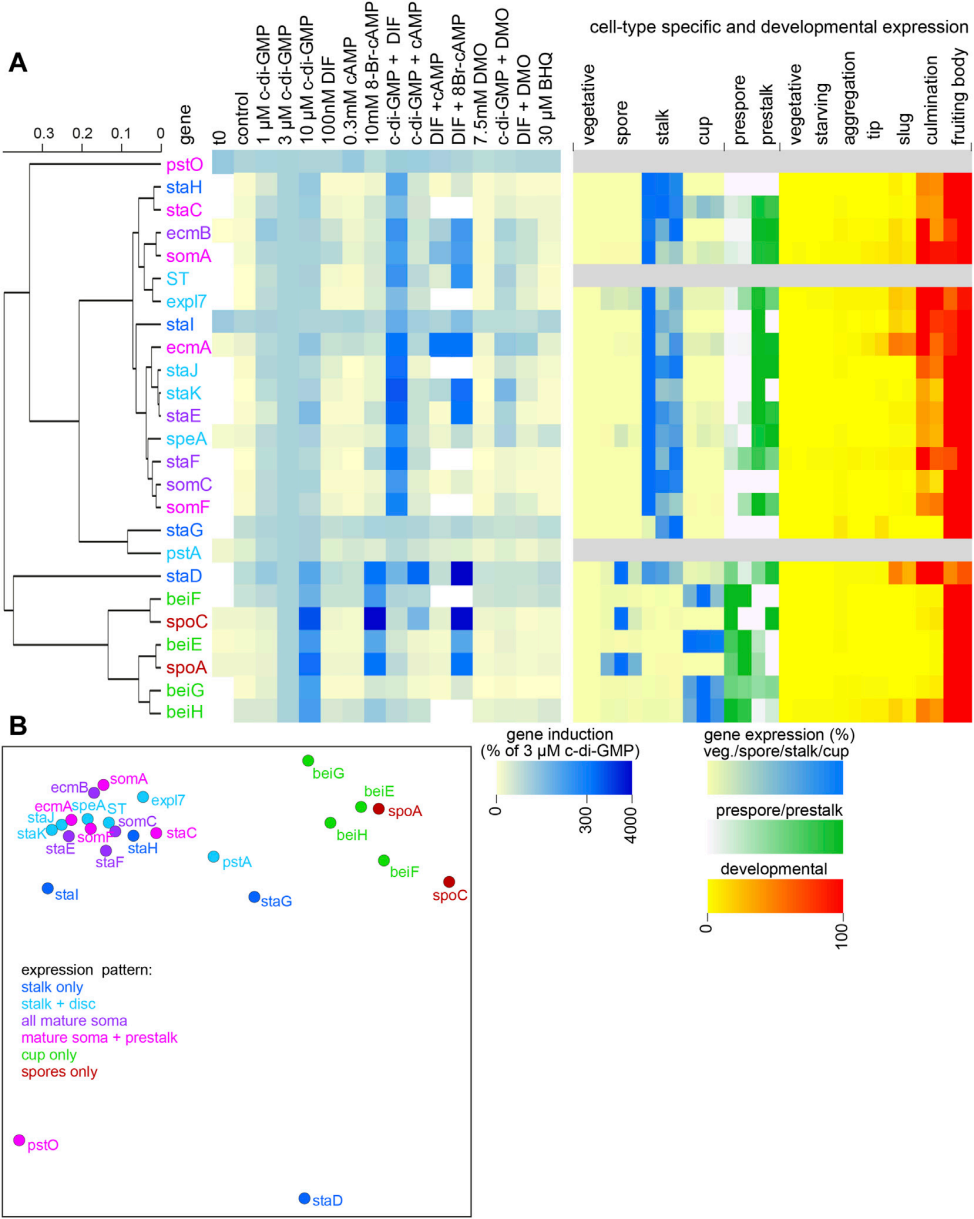
Cluster analysis of gene induction data. **(A)** *. Hierarchical tree.* The averaged gene induction data of Figure 2, expressed as percentage of induction by 3 µM c-di-GMP (also for *staD*) were subjected to hierarchical clustering in Orange (Demsar et al., 2013). Distances between genes were determined by Pearson correlation of their responses to the different stimuli and a hierarchical tree was inferred from the distance matrix by average linkage. **(A)** The tree is annotated with a heatmap of the averaged gene induction responses and a heatmap of the cell-type specific and developmental expression of the genes derived from two or three replicate experiments. **(B)**. *Multidimensional scaling.* The induction data were also subjected to multidimensional scaling, an iterative method that yields a two-dimensional projection of points, which minimizes the distances between them. Cluster analysis based on gene induction data expressed as fold-change relative to control (no addition) is shown in Supplementary Figure S7.

To identify gene sets that respond similarly to external stimuli, we performed hierarchical cluster analysis on the relative expression levels of the genes in response to the added stimuli. Separate analyses were performed on the data expressed as percentage of induction by 3 µM c-di-GMP (Figure 3) and as fold-change relative to control (unstimulated) expression (Supplementary Figure S7).

The responses to gene induction by stimuli and the developmental- and cell type specific expression of the genes were plotted onto the hierarchical trees as heatmaps (Figure 3A centre and right panel). While the hierarchical trees based on either percentage induction or on fold-change show a different topology, both show a single cluster that contains all spore and cup-only genes, a large cluster that contains all but four of the stalk-expressed genes, a small cluster containing *staG* and *pstA* and separate branches for *pstO* and *staD* each. The induction heatmap next to the tree shows that the *staG*, *pstA* and *pstO* show little response to any of the added stimuli. The induction profile of the stalk-specific gene *staD* is more similar to that of the spore and cup cells and this gene is also unusual in requiring cAMP for efficient induction by c-di-GMP.

The cup + spore set is characterized by upregulation by 10 µM c-di-GMP and by 8Br-cAMP and by downregulation of c-di-GMP induction by DIF-1 and DMO. The large cluster of stalk genes is defined by upregulation by 1–10 µM c-di-GMP and synergy between c-di-GMP and DIF-1. Multidimensional scaling, another method of identifying clusters of related genes (Figures 3B; Supplementary Figure S6B) shows a similar clustering of spore and cup genes, four outgroup genes and a large cluster of the remaining stalk genes. Apart from the finding that three of the four stalk-only genes are outliers to the large cluster of stalk-expressed genes, there is no notable clustering of stalk genes that are variably expressed in other somatic cells or in prestalk cells. Note that *staD* also shows expression in prespore, spore and cup cells (Figure 3A, right panel). Its outlier status, inclusive of the fact that, like cup cells and spores, its induction requires rather high c-di-GMP concentrations (Figure 2A), may therefore be due to *staD* being regulated by mixed signals.

## Discussion

### Somatic Cell Fate Is Likely Determined Late in Development

The paucity of marker genes for spore, stalk, basal disc and cup cells led us to perform a transcriptome analysis of purified spore, stalk and cup cells (Kin et al., 2018). Basal disc cells could not be purified, since they are too firmly attached to the stalk. Here and in the parent study we validated the expression patterns of highly cell-type enriched and well-expressed genes that were preferentially also conserved across all taxon groups of Dictyostelia. Many well-expressed spore- and stalk-specific genes encode matrix proteins with a signal peptide, cellulose binding domains or repeats. They are members of families that underwent extensive gene gain and loss and are mostly not identifiable as orthologs across species. Conserved genes with high and specific expression were relatively rare. None of the eight validated cup-specific genes *beiA* to *beiH* were conserved outside group 4 (this study and (Chen et al., 2017)), likely also reflecting that cup cells are an evolutionary novelty of group 4 (Kin et al., 2021; Schilde et al., 2014). Deeply conserved spore-specific genes with conserved spore specificity such as *spoA-spoC* were easier to find and five deeply conserved stalk-enriched genes were found, of which *expl7*, *somA* and *somD* are also stalk-specific across taxon groups. *Expl7* was already identified earlier as a stalk gene regulated by the transcription factor STATa (Ogasawara et al., 2009). Cluster analysis of gene induction (Figure 3) showed that *expl7* and *somA* are part of a large clade of similarly regulated stalk genes and may therefore be representative stalk markers for deep evolutionary studies. Otherwise, the investigated set contains both cup and stalk markers that are widely conserved within group 4, such as *staF, staI, staK, somB, beiG* and *beiH* as well as the traditional markers *ecmA* and *ecmB* (Jermyn and Williams, 1991), which result from a gene duplication early in the group 4 lineage (Supplementary Figure S3).

While selected for high cell-type enrichment, most stalk genes were also expressed in the basal disc and cup cells in promoter-*lacZ* studies (Figure 1) (Kin et al., 2018). For the basal disc cells this was expected since they could not be separated from the stalks and have a similar phenotype as stalk cells. However, it is less clear why cup cells, which remain amoeboid throughout development should share so many genes with stalk cells. As an alternative to a view that the group 4 specific cup and disc cells are an entirely novel cell type, the cup and disc expression of most stalk genes suggests the presence of an ancestral somatic cell pool, from which at first the stalk and later the cup and disc cells evolved.

The cup genes identified from purified cup cells are specific to cup cells and expressed late in fruiting body formation when spores are maturing (Kin et al., 2018). The cup expression from stalk genes, such as *ecmA* and *ecmB*, is already well visible at early culmination and can be retraced to expression in anterior-like cells (ALCs) in slugs. Recently, a transcription factor, *cdl1a*, was identified that is essential for late cup gene expression and cup differentiation (Kin et al., 2021). Expression in ALCs and early cup regions from the stalk gene *ecmA* and *ecmB* still occurred in *cdl1a* null mutants, but the expressing cells became dispersed in the spore mass and later formed a side branch to the main stalk with a stalk and spore head. This indicates that the *ecmA* or *ecmB* expressing cup cells were not committed to cup differentiation until the *cdl1a* regulated cup genes were expressed. This favours a hypothesis that the prestalk and ALC populations represent undifferentiated soma, from which terminal cell types only become specified at a later stage. Species outside group 4 often split off many side branches from the main cell mass during fruiting body formation. It is plausible that these side branches also originate from an unspecified soma population.

### C-Di-GMP Is a Stalk Inducer Because Its Synthesis and Target Are Prestalk-Restricted

C-di-GMP was put forward as an inducer of stalk differentiation because mutants that lack its synthetic enzyme DgcA failed to form stalks (Chen and Schaap, 2012). However, comparison of wild-type and *dgcaˉ* transcriptomes indicated that apart from stalk genes, cup genes were not expressed in *dgcaˉ* either (Chen et al., 2017). Here, we found that c-di-GMP also upregulates spore gene expression, but similar to cup genes at about 30-fold higher concentrations than needed for stalk gene induction. C-di-GMP hyperactivates the adenylate cyclase AcaA, which in turn activates PKA resulting in stalk cell differentiation (Chen et al., 2017). Spore maturation also requires PKA (Hopper et al., 1993), which is in prespore cells achieved by activation of the adenylate cyclases ACR and ACG (Soderbom et al., 1999; Alvarez-Curto et al., 2007) and inhibition of the cAMP phosphodiesterase RegA (Anjard and Loomis, 2005). Cup gene expression is inducible by the PKA agonist 8Br-cAMP (Chen and Schaap, 2012) and therefore also likely to require PKA activation. Since *dgcA* is only expressed in the anterior prestalk cells (Chen and Schaap, 2012) and *acaA* is preferentially expressed at the slug tip (Verkerke-van Wijk et al., 2001), c-di-GMP activates formation of the stalk at the slug tip. However, since AcaA is not restricted to tip cells, exogenously applied c-di-GMP is likely to induce some PKA activity in pre-cup and pre-spore cells as well, although 8Br-cAMP is a more effective inducer than c-di-GMP, while for stalk genes the reverse is true.

### Missing Signals for Somatic Cell-type Specialization

Due to the localization of DgcA and AcaA in prestalk and tip cells respectively, c-di-GMP activation of PKA specifically activates stalk formation. However, as spore-, cup- and likely also basal disc maturation require PKA as well, this does not explain how these 4 cell types were fate-mapped in the first place. Prespore differentiation is initiated by activation of cAMP receptors by secreted cAMP (Schaap and Van Driel, 1985; Wang et al., 1988b). This response, as well as the role of PKA in spore and stalk maturation, is deeply conserved in Dictyostelia (Ritchie et al., 2008; Kawabe et al., 2009; Kawabe et al., 2015).

DIF-1 was identified as a secreted signal that induced stalk-like cells *in vitro* (Morris et al., 1987)*.* However, mutants without DIF-1 synthetic enzymes, such as StlB, still formed stalks, which were however relatively thin and lacked the basal disc (Saito et al., 2008). Outside group 4, DIF-1 was only detected in its sister species *P. violaceum* (Kay et al., 1992), but this species has no basal disc and here deletion of *stlB* results in thicker rather than thinner stalks (Narita et al., 2020). An evolutionary conserved signal for stalk cell specification is therefore still missing. If anything, DIF-1 negatively regulates cup gene expression (Figure 2) and without other candidates, a cup-specifying signal is therefore also missing. Species across all four dictyostelid taxon groups form stalks, while cup and disc cells are evolutionary novelties of group 4, as is the emergence of DIF-1 signalling in the group 4 lineage. While the missing cup signal may also be unique to the group 4 lineage, the search for a stalk-specifying signal would benefit from a broader study across Dictyostelia.

### Cluster Analysis of Gene Regulation Does Not Identify Similarly Expressed Soma

Based on *in vivo* expression patterns, we roughly subdivided genes in stalk-, cup- and spore only genes, and in stalk + disc, stalk + disc + cup (mature soma) and mature soma + prestalk/ALC expressed genes (Figure 2). At least four representative genes for each somatic cell type were tested for regulation *in vitro* by signals or combinations of signals known to induce or regulate stalk-like gene expression, such as DIF-1, c-di-GMP, 8Br-cAMP and cAMP, as well as the weak acid DMO and the Ca^2+^-ATPase inhibitor, BHQ, which were reported to promote or mediate effects of DIF-1 by decreasing cytosolic pH or raising Ca^2+^, respectively (Gross et al., 1983; Wang et al., 1990; Kubohara and Okamoto, 1994; Schaap et al., 1996). To identify commonalities between the regulation of the 25 tested genes by the 15 stimulation regimes, we subjected the induction data to hierarchical clustering. The analysis yielded a single cluster of the spore and cup only genes, which are united by their strong up-regulation by high (10 µM) c-di-GMP only and downregulation of the c-di-GMP induction by DIF-1. All but four of the stalk-expressed genes form a single cluster that is united by strong upregulation by 1–10 µM c-di-GMP, synergistic or additive upregulation by combined DIF-1 and c-di-GMP, modest upregulation by DIF-1 or BHQ and a modest additive effect of DMO on upregulation by DIF-1. There was no obvious clustering within this set of genes expressed in stalk + disc, mature soma or mature soma and prestalk, indicating that none of the tested signals selectively regulated expression in either stalk, disc or cup cells. In other words, genes that are variably expressed in what were considered to be different somatic cell types appear to be regulated by the same signals, so either the cell types are not different i.e. they all belong to the same somatic (non-prespore) cell pool or they are not specified by currently known signals.

The remaining five genes *staI, staD, staG, ecmA_pstO* and *ecmA_pstA* were all outliers that show little (*ecmA_pstA* and *staG*) or no upregulation by c-di-GMP or by DIF-1. As discussed above, for *staD* this was likely due to limited stalk specificity. *PstA* and *pstO* are previously characterized fragments of the *ecmA* promoter that are respectively expressed in the front and back of the slug prestalk region (Early et al., 1993). Their regulation does not add up to that of the DIF-1 and c-di-GMP activated full *ecmA* promoter, indicating that results obtained with these markers should be interpreted with caution. *StaI* and *staG* represent two out of the four tested stalk-only genes. Their lack of upregulation by DIF-1 and/or c-di-GMP demonstrates that at least one other signal apart from these two can induce stalk-specific gene expression.

Overall, the present study highlights the need for a renewed search into the signals and pathways that control cell-type specialization in Dictyostelia and provides some of the tools to do so in an evolutionary context.

## Data Availability

The original contributions presented in the study are included in the article/Supplementary Material, further inquiries can be directed to the corresponding authors. All plasmid constructs were deposited in the Dicty Stock center http://dictybase.org/StockCenter/StockCenter.html.
