# Supplementary material for: Novel RNAseq-Informed Cell-type Markers and Their Regulation Alter Paradigms of Dictyostelium Developmental Control
Source: Front Cell Dev Biol. 2022 May 5;10:899316. doi: 10.3389/fcell.2022.899316 (PMC9117722; doi:10.3389/fcell.2022.899316)
Supplement: Supplementary file 3 [file Presentation1.PDF]

Supplementary Figures S1-S7 and supplementary Table S1

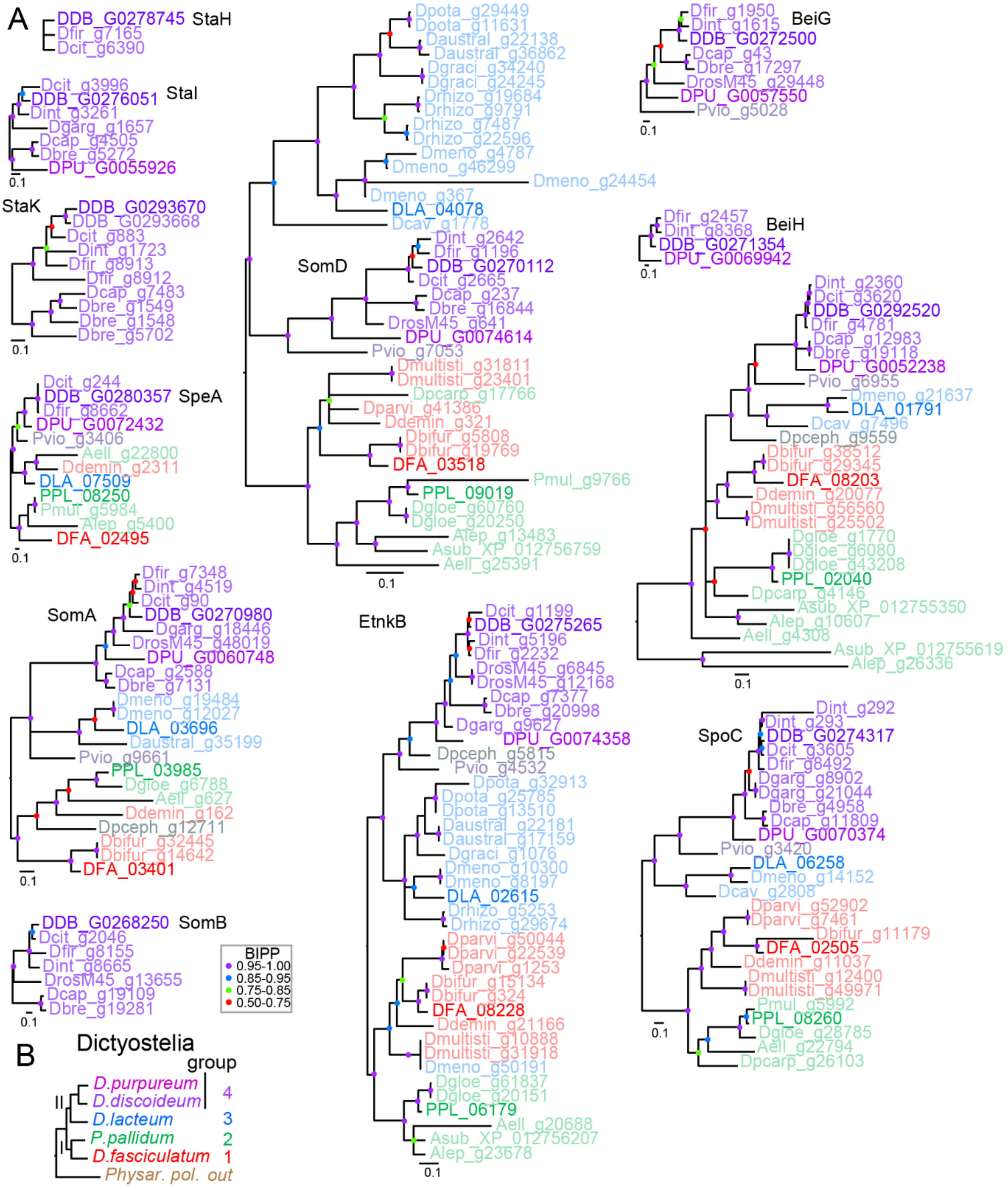

Figure S1. Evolutionary conservation of newly analysed cell type marker genes

A. Gene conservation. For the cell type marker genes that were analysed in figure 1, closest relatives were identified by BLASTp in a protein sequence library compiled from 47 annotated genomes and transcriptomes, representative of all major and minor taxon groups of Dictyostelia. The locus tags of the genes are colour coded according to the taxon group to which the host species belongs (B) with the more intense colours representing the well annotated *D. discoideum*, *D. purpureum*, *D. lacteum*, *P. pallidum* and *D. fasciculatum* genomes. Species belonging to minor taxon groups are colour coded in grey. Protein sequences were aligned

with Clustal Omega (Sievers and Higgins, 2014) with five combined iterations and phylogenies were inferred using MrBayes 3.2 (Ronquist and Huelsenbeck, 2003). Posterior probabilities (BIPP) of the nodes are indicated by coloured dots. Full species names and Bioproject or Assembly IDs of the genomes and transcriptomes that were used are listed in SupData1_Genes&Species.xlsx.

B. Dictyostelid phylogeny. Phylogeny of the five taxon-group-representative *Dictyostelium* species for which developmental- and cell type specific transcriptome data are shown in Figure 1B. The phylogeny was inferred from 30 concatenated proteins and rooted on the non-dictyostelid Amoebozoan *Physarum polycephalum* (Romeralo et al., 2013).

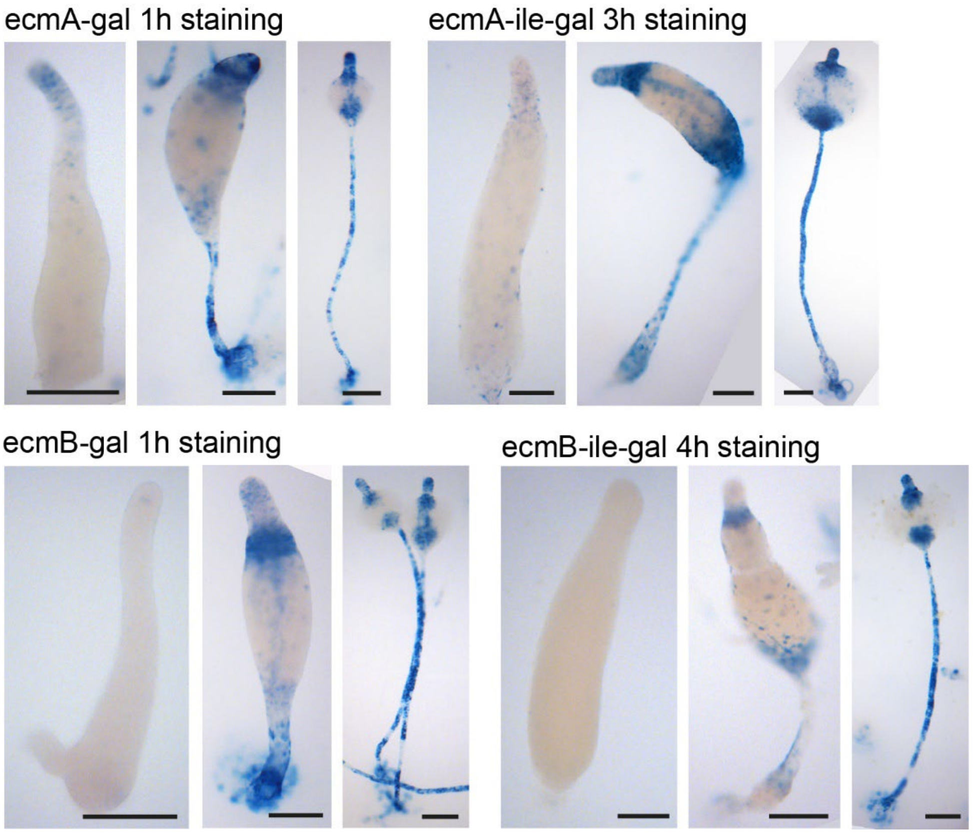

Figure S2. Standardized staining of cells transformed with *ecmA*- and *ecmB-lacZ*
Ax2 cells transformed with *ecmA-lacZ*, *ecmA-ile-lacZ* , *ecmB-lacZ* and *ecmB-ile-lacZ* (Detterbeck et al., 1994; Jermyn and Williams, 1991) were incubated on nitrocellulose filters supported by non-nutrient agar until migrating slugs, mid-culminants and fruiting bodies were formed. After fixation, the structures were stained with X-gal for the period required to strongly but not overstain the culminants and fruiting bodies. Bar: 100 µm. Note that the poor expression in slugs is not a consequence of β-galactosidase accumulating in the later stages, since the unstable ile-gal forms (Detterbeck et al., 1994) also show poor staining in slugs compared to fruiting bodies.

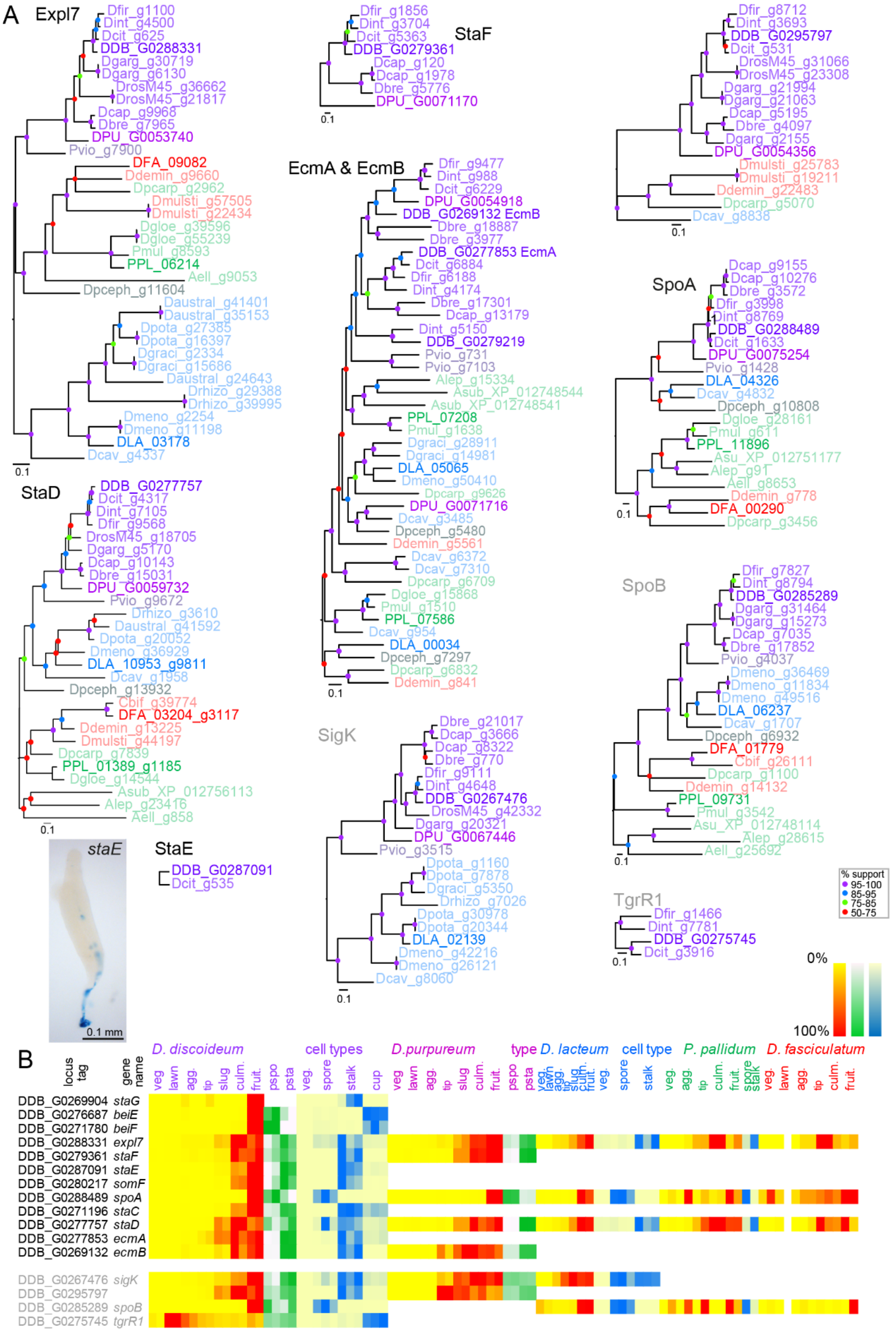

Figure S3. Conservation and transcription profiles of previously described marker genes

A. Gene conservation. For the gene induction experiment presented in figure 2, we used additional cell type markers from an earlier study (Kin et al., 2018), the DIF-inducible markers *ecmA* and *ecmB* (Jermyn et al., 1987) and two markers identified in a RNAseq study of mutants that cannot synthesize c-diGMP (Chen et al., 2017) . These and other markers from the same studies that were validated using promoter-lacZ constructs were analysed for evolutionary conservation as in Fig. S1. Replicate studies of staE-lacZ showed expression in the basal disc of culminants (image), which was not reported in the earlier study (Kin et al., 2018).

B. Transcriptomics. The developmental time courses and cell type specificity data of the markers were retrieved from published RNAseq experiments as described in the legend to Figure 1. When known, gene names are also listed. Identifiers in grey text represent genes not otherwise used in this study.

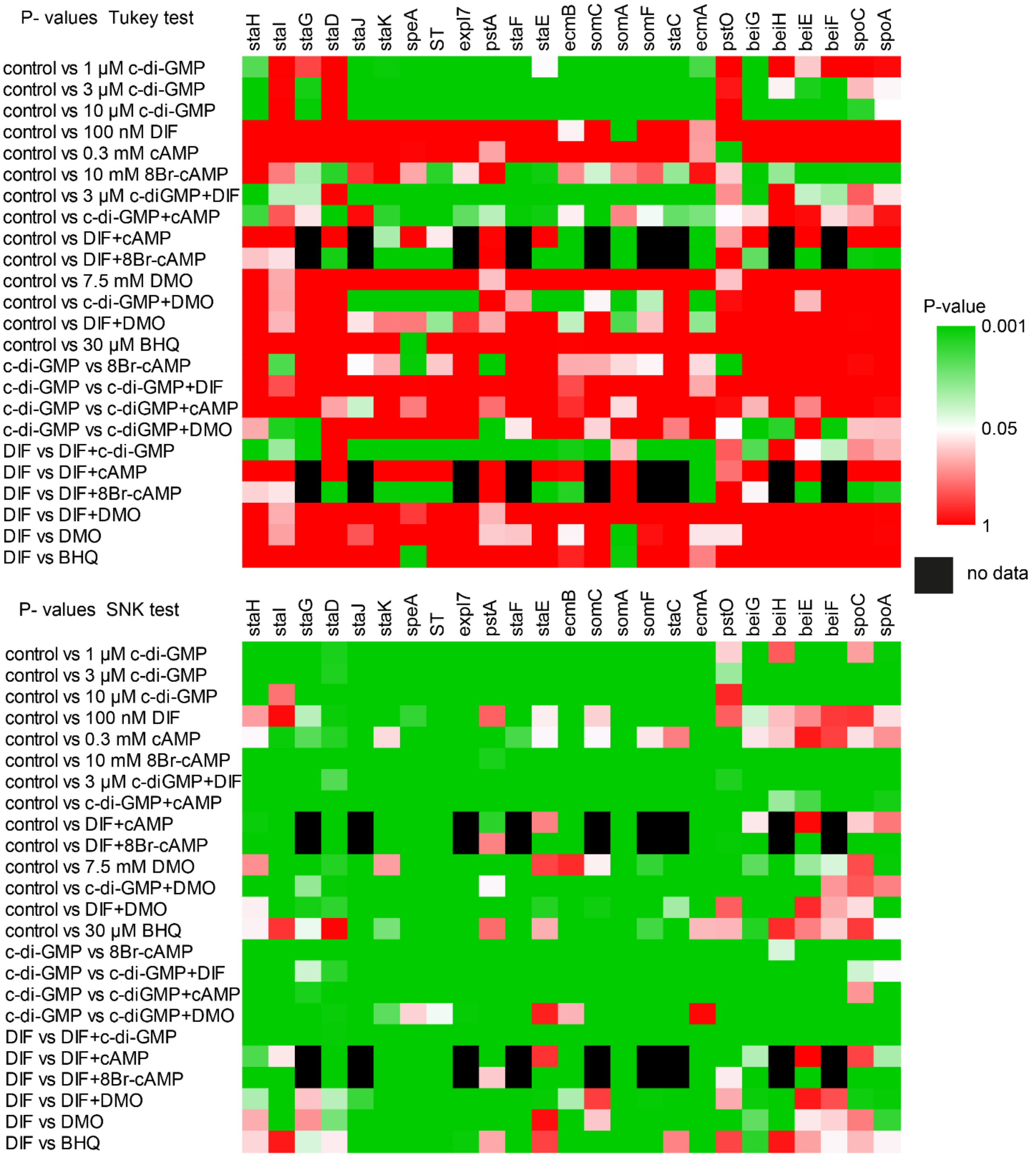

Figure S4. P-values for pair-wise comparison of the differential effects of stimuli on gene induction
The data describing the effects of 15 stimulation regimens on 25 genes that are presented in figure 2 were subjected to Kruskal-Wallis ANOVA on ranks to identify significant differences between treatments on each of the genes. The stringent Tukey test and less stringent Student-Newman-Keuls (SNK) test were used to calculate P-values for the null hypothesis of no difference between treatments. The full analysis of 105 pairwise comparisons is listed with the experimental data for each gene in SupData2_Induction.xlsx. Biologically relevant comparisons are shown here as heatmaps of the P-values obtained with either test. Black squares represent experiments that were only performed once or not at all. Combined treatments used 3 µM c-di-GMP and the same concentrations for the other compounds as used for single treatments.

fold change over control

gene	1 µM c-di-GMP	3 µM c-di-GMP	10 µM c-di-GMP	100 nM DIF	0.3 mM cAMP	10 mM 8-Br-cAMP	c-di-GMP + DIF	c-di-GMP+cAMP	DIF+cAMP	DIF+8-Br-cAMP	7.5 mM DMO	c-di-GMP+DMO	DIF +DMO	30 µM BHQ
staH	24	52	59	1.4	2.0	7.4	91	29	1.1	9.7	1.4	18	1.3	2.0
staI	1.1	1.0	1.0	1.0	1.0	0.8	1.9	1.1	1.0	1.5	0.8	0.8	0.8	1.0
staG	1.3	1.4	1.4	0.9	1.1	1.4	1.3	1.3	1.1	1.4	0.9	0.9	0.9	1.1
staD	1.4	1.4	2.3	0.9	0.8	5.4	2.0	11	0.8	47	0.7	0.8	0.8	1.0
staJ	28	29	25	5.4	0.2	6.5	142	7.2			0.6	14	6.7	3.3
staK	373	482	462	17	8.4	96	2350	221	186	699	16	792	82	20
speA	5.7	6.9	6.8	1.1	1.5	2.1	17	4.1	1.4	5.1	1.3	7.0	2.1	4.2
ST	73	95	99	8.4	5.9	42	212	85	72	276	2.4	90	34	1.7
expl7	71	91	89	6.6	3.6	38	137	53			2.1	80	29	3.2
pstA	2.6	3.0	3.1	1.0	1.4	1.2	2.7	1.7	1.2	1.1	0.5	1.1	0.5	1.1
staF	122	152	148	20	0.9	126	466	95			-1.1	25	12	4.6
staE	8.3	20	30	1.1	0.8	12	170	14	1.0	62	1.1	21	1.7	1.0
ecmB	16	13	13	8.1	2.1	5.3	31	7.1	15	30	1.0	13	8.8	2.6
somC	129	157	151	7.5	3.9	26	342	40	3.5	36	2.3	20	2.4	7.4
somA	11	12	12	10	3.0	5.9	25	5.9	16	23	0.7	9.5	7.9	1.6
somF	9.5	16	14	3.0	1.2	4.7	45	6.7			0.9	8.8	6.3	1.2
staC	8.8	11	12	1.8	1.0	5.9	17	6.1			0.8	3.2	1.4	2.1
ecmA	9.3	15	21	3.2	8.6	6.0	60	11	71	77	0.7	16	13	1.7
pstO	1.0	1.1	1.0	1.0	1.3	0.8	1.2	1.2	1.1	1.0	0.8	0.9	1.0	1.0
beiG	13	45	97	1.2	1.3	22	23	4.6	1.1	20	0.8	2.3	0.8	0.9
beiH	1.2	3.1	5.9	1.1	1.2	4.1	2.8	1.5			0.6	1.4	0.7	1.0
beiE	3.9	15	31	1.1	1.2	26	8.1	2.1	1.1	27	0.9	4.7	1.1	1.3
beiF	1.4	1.7	2.9	1.0	1.1	3.7	1.6	1.5			1.0	1.1	1.0	1.1
spoC	3.0	8.4	141	1.9	1.2	419	6.5	19	2.7	420	1.7	2.0	-0.4	0.6
spoA	1.5	5.7	28	1.1	1.1	21	4.3	1.5	1.0	23	0.7	1.1	0.6	0.9

between treatments: first over second

8BrcAMP/c-di-GMP	c-di-GMP+DIF/c-di-GMP	DIF+c-di-GMP/DIF	c-di-GMP+DMO/c-di-GMP	DIF+DMO/DIF	c-di-GMP+cAMP/c-di-GMP	DIF+cAMP/DIF	DIF+8Br-cAMP/DIF	gene
0.1	1.7	65	0.3	0.9	0.6	0.8	6.9	staH
0.8	1.8	1.9	0.8	0.8	1.1	1.0	1.5	staI
1.0	1.0	1.4	0.7	1.0	0.9	1.2	1.5	staG
3.8	1.4	2.2	0.5	0.9	7.7	0.9	53	staD
0.2	4.9	26	0.5	1.2	0.2			staJ
0.2	4.9	139	1.6	4.8	0.5	11	41	staK
0.3	2.5	15	1.0	1.8	0.6	1.3	4.5	speA
0.4	2.2	25	0.9	4.1	0.9	8.6	33	ST
0.4	1.5	21	0.9	4.4	0.6			expl7
0.4	0.9	2.7	0.4	0.5	0.6	1.2	1.1	pstA
0.8	3.1	24	0.2	0.6	0.6			staF
0.6	8.4	151	1.0	1.5	0.7	0.9	55	staE
0.4	2.4	3.8	1.0	1.1	0.5	1.8	3.8	ecmB
0.2	2.2	46	0.1	0.3	0.3	0.5	4.8	somC
0.5	2.1	2.5	0.8	0.8	0.5	1.6	2.3	somA
0.3	2.7	15	0.5	2.1	0.4			somF
0.5	1.5	10	0.3	0.8	0.5			staC
0.4	3.9	19	1.1	3.9	0.7	22	24	ecmA
0.8	1.1	1.2	0.9	1.0	1.1	1.1	1.0	pstO
0.5	0.5	20	0.1	0.7	0.1	0.9	17	beiG
1.3	0.9	2.7	0.5	0.7	0.5			beiH
1.7	0.5	7.6	0.3	1.0	0.1	1.0	25	beiE
2.2	0.9	1.5	0.7	1.0	0.9			beiF
49.7	0.8	3.5	0.2	-0.2	2.2	1.4	224	spoC
3.7	0.8	4.0	0.2	0.6	0.3	0.9	21	spoA

0 200 2400 **95** P-val<0.05 Tukey *8.6* P-val<0.05 SNK 0 1 30 >30

Figure S5. Fold-change differences between treatments annotated with P-values

The table lists the numerical values of the fold-change over control values (left panel) and fold-changes between different treatments (right panel). The values are presented in different fonts to reflect whether the difference between treatments is significant (P<0.05) as assessed by ANOVA on ranks with P-values calculated by either the stringent Tukey test (bold black or white font) or the less stringent SNK test (italic red font). The extent of fold-change is further indicated as a yellow-blue heatmap in the left panel and a blue to red heatmap in the right panel, where a blue background marks a reduced effect and a red background an increased effect of the first over the second treatment, shown at the top of each column. Black boxes represent absent data. Note that small fold-changes can be statistically significant when there is little variation between individual experiments, while large fold-changes may not reach significance because the absolute value of the change varies strongly between experiments. We therefore also show the data and their analysis in Supdata2_induction.xlsx.

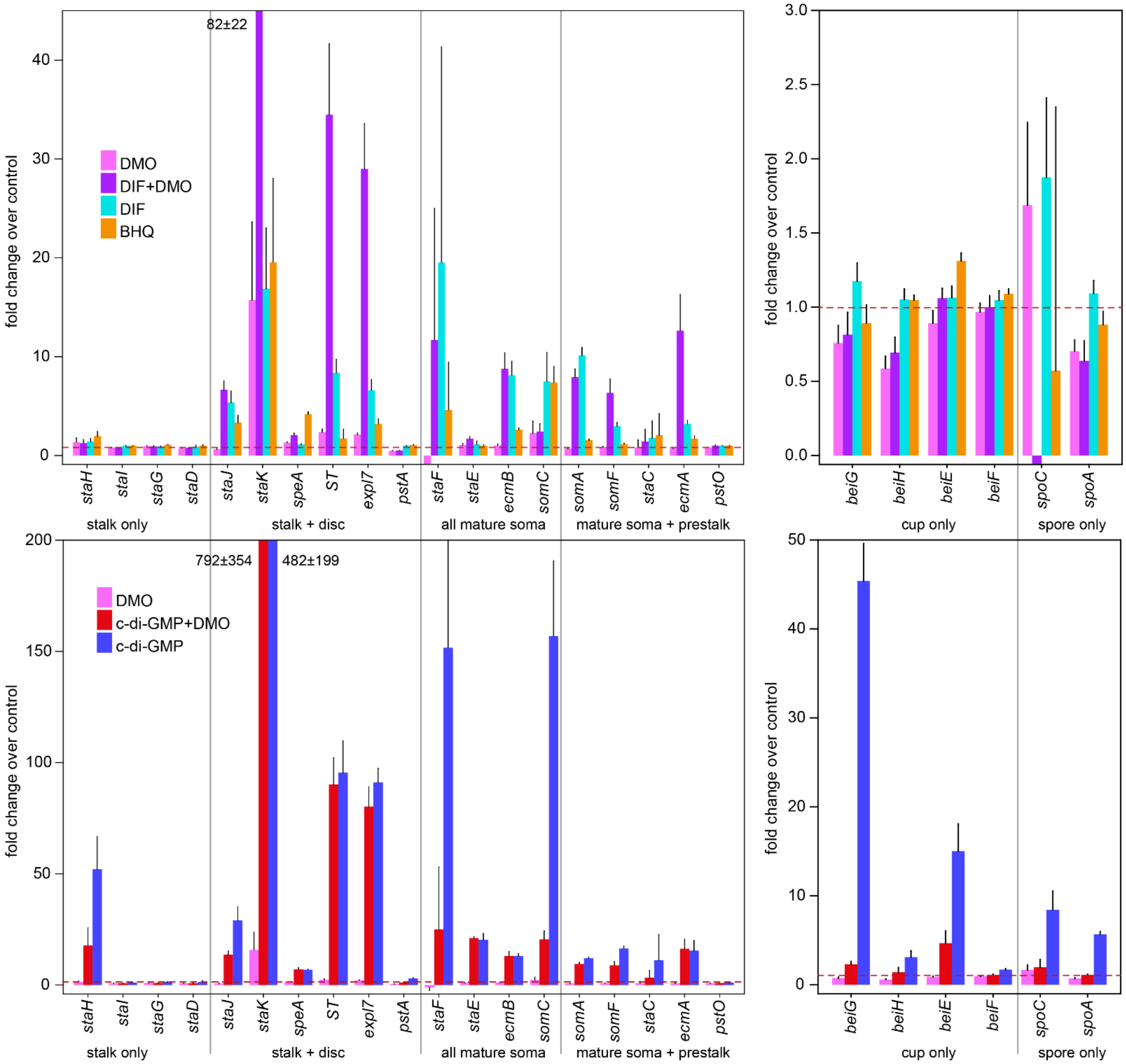

Figure S6. Gene induction expressed as fold-change of control
For a subset of the gene induction data presented in Figure 2 where relatively small effects of stimuli were observed, data are re-plotted here as means and SE of fold-change over control (no addition). The red dashed line marks the control value of 1.

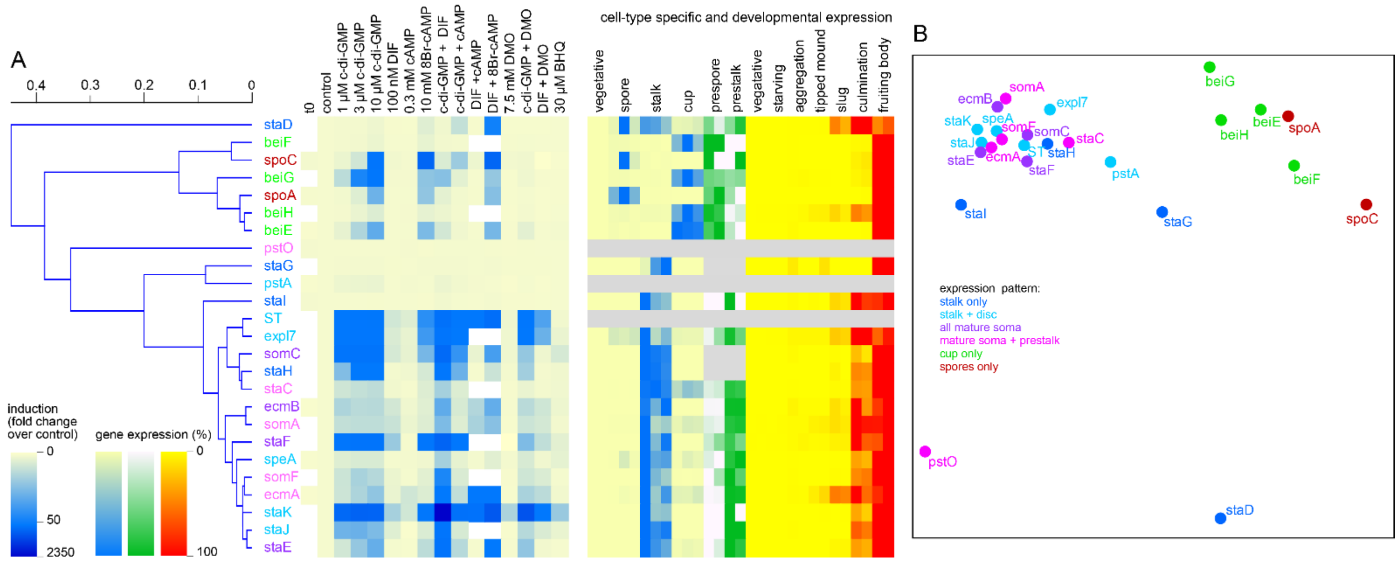

Figure S7. Hierarchical clustering of gene induction data relative to fold change

A. Hierarchical tree. The averaged data for induction of 25 genes by 15 single or combined stimuli as presented in figure 2 are expressed here as fold-change relative to control and subjected to hierarchical clustering as described for figure 3. The hierarchical tree is combined with a heatmap of the gene induction responses as fold-change over control and a heatmap of the cell-type specific and developmental expression of the genes.

B. Multidimensional scaling. The induction data relative to fold-change were also used to cluster the genes by multidimensional scaling as in figure 3.

Table S1: Oligonucleotide primers used in this work

Gene ID	coordinates from ATG	5' gene	primer name	Oligonucleotide sequence
278745	-881_+15	-842	278745-Fw	CCC**TCTAGA**GATTCAATAATTAATTTTATTAATATTAC
			278745-Rv	CCC**AGATCT**TGAATAAATTTTCATTTTTAATTTCCT
276051	-961_+24	-937	276051-Fw	CCC**TCTAGA**TTCTTACTCATATTCTTTACTCAT
			276051-Rv	CCC**AGATCT**TTCTTGATTTTGAAAGTTTGACAT
272500	-3120_+33	-3081	272500-Fw	CCC**TCTAGA**TGATGGGTTTCTTAAAATGAATGATGGTTTATTTTGCAT
			272500-Rv	CCC**AGATCT**AGTACTCTCTGAATAATTACTTTTGTTATCCAT
271354	-990_+27	-963	271354-Fw	CCC**TCTAGA**ATTATCTAAAATCATTGAAATTCCCAT
			271354-Rv	CCC**GGATCC**ACAAATTAATTTAATTATTAATTTCAT
271972	-1446_+18	-1410	271972-Fw	CCC**GCTAGC**GGTTCATTATATAAAATTGCTGTAGTTAAAC
			271972-Rv	CCC**AGATCT**GAGAATTATTTTATTCATTTTTATTTTATTTTATTTTAATTTG
293670	-1336_+21	-1306	293670-Fw	CCC**TCTAGA**ACACTATTTATTAAAGTGTCAAAAAATTAA
			293670-Rv	CCC**AGATCT**GTCATATTCATTTTGTGACAT
280357	-983_+39	-3074	280357-Fw	AA**TCTAGA**GATTCAATTTTAAAAATTATCACCAGT
			280357-Rv	AA**AGATCT**TGATTTCTTTTTCTTTGAAGAAAGAAACAT
270980	-1172_+27	-1142	270980-Fw	CCC**TCTAGA**CAGATTGATAAGTGCTTTTCTTCTTGACAT
			270980-Rv	CCC**AGATCT**TTGTTGTTGTTGTTGTGGCTGATCCAT
268250	-1691_+36	-1658	268250-Fw	CCC**TCTAGA**AATACACCACATCTTCATAAAAATTCTTATTAA
			268250-Rv	CCC**AGATCT**TGAAATTAAAATTAAATTATTAATTTTCATTTTCAT
280277	-700_+27	-685	280277-Fw	CCC**TCTAGA**GTTTTCTTTGCATAAATAGTATAAAACCCT
			280277-Rv	CCC**GGATCC**ACTTAATAAAATAAGAATTGATCTCAT
270112	-3167_+29	-3150	270112-Fw	CCC**GCTAGC**GATGTAATTGATTTCATCTTTACACTT
			270112-Rv	CCC**AGATCT**TCTTTACTTATTTGATTTTGTTGTTCCAT
286649	-1161_+3	-1150	286649-Fw	CCC**TCTAGA**AGGACCTACATTTTTTAAATTGATTG
			286649-Rv	CCC**AGATCT**CATTTTTCTTTTTTTTTTTTAATAAATAAATATAAATTTTAG
275265	-1130_+33	-1094	275265-Fw	CCC**AGATCT**TGGACCTGGATAAATAATATCTTCAAACTCGTTCAT
			275265-Rv	CCC**TCTAGA**AGGGTGGGGAGTGCAGGTGTTTTAGTATTTTAG
292520	-2384_+18	-3174	292520-Fw	CCC**TCTAGA**ATTAAAATAAAAACTATGAATAATATGAGG
			292520-Rv	CCC**AGATCT**CATTATTGGTGAATCCATTGT
274317	-929_+21	-884	274317-Fw	CCC**TCTAGA**ACTCCAACTTCATCCTCCTCAAAT
			274317-Rv	CCC**AGATCT**TCTATTTTTAACTGATTCCATAATTTGGTT

Genes are listed by the Dictybase geneID from which the prefix DDB_G0 was deleted. The coordinates of the amplified 5'-intergenic regions are shown in column 2, with the start ATG at +1. The distance of the upstream gene from the start codon is shown in column 3 and the names and sequences of the PCR primers in columns 4 and 5. The restriction sites used for cloning are indicated in bold text.

SUPPLEMENTARY REFERENCES

Chen, Z. H., Singh, R., Cole, C., Lawal, H. M., Schilde, C., Febrer, M., Barton, G. J. and Schaap, P. (2017). Adenylate cyclase A acting on PKA mediates induction of stalk formation by cyclic diguanylate at the Dictyostelium organizer. *Proc Natl Acad Sci U S A* **114**, 516-521.

Detterbeck, S., Morandini, P., Wetterauer, B., Bachmair, A., Fischer, K. and MacWilliams, H. K. (1994). The 'prespore-like cells' of *Dictyostelium* have ceased to express a prespore gene: Analysis using short-lived beta-galactosidases as reporters. *Development* **120**, 2847-2855.

Jermyn, K. A., Berks, M., Kay, R. R. and Williams, J. G. (1987). Two distinct classes of prestalk-enriched mRNA sequences in Dictyostelium discoideum. *Development* **100**, 745-755.

Jermyn, K. A. and Williams, J. G. (1991). An analysis of culmination in Dictyostelium using prestalk and stalk-specific cell autonomous markers. *Development* **111**, 779-787.

Kin, K., Forbes, G., Cassidy, A. and Schaap, P. (2018). Cell-type specific RNA-Seq reveals novel roles and regulatory programs for terminally differentiated Dictyostelium cells. *BMC Genomics* **19**, 764.

Romeralo, M., Skiba, A., Gonzalez-Voyer, A., Schilde, C., Lawal, H., Kedziora, S., Cavender, J. C., Glockner, G., Urushihara, H. and Schaap, P. (2013). Analysis of phenotypic evolution in Dictyostelia highlights developmental plasticity as a likely consequence of colonial multicellularity. *Proc Biol Sci* **280**, 20130976.

Ronquist, F. and Huelsenbeck, J. P. (2003). MrBayes 3: Bayesian phylogenetic inference under mixed models. *Bioinformatics* **19**, 1572-1574.

Sievers, F. and Higgins, D. G. (2014). Clustal omega, accurate alignment of very large numbers of sequences. *Methods in molecular biology* **1079**, 105-116.
